# Supplementary material for: Waning of Humoral Immunity and the Influencing Factors after BNT162b2 Vaccination: A Cohort Study with a Latent Growth Curve Model in Fukushima
Source: Vaccines (Basel). 2022 Nov 25;10(12):2007. doi: 10.3390/vaccines10122007 (PMC9782062; doi:10.3390/vaccines10122007)
Supplement: Supplementary file 1 [file vaccines-10-02007-s001.zip › vaccines-2017422-supplementary.pdf]

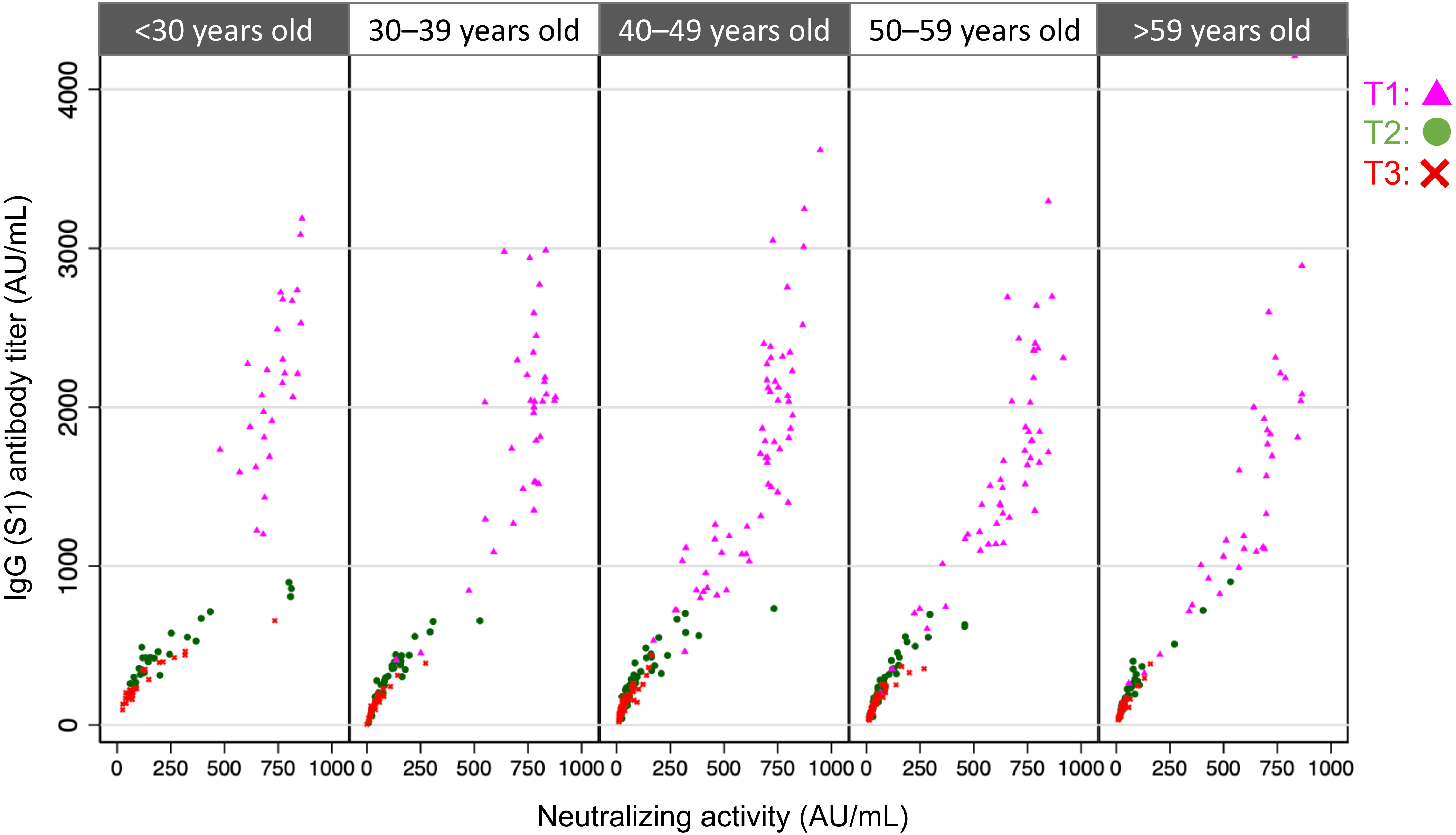

Figure S1. Comparison between IgG antibody titer and neutralizing activity in each age category.
T1; Time 1, T2; Time 2, T3; Time 3

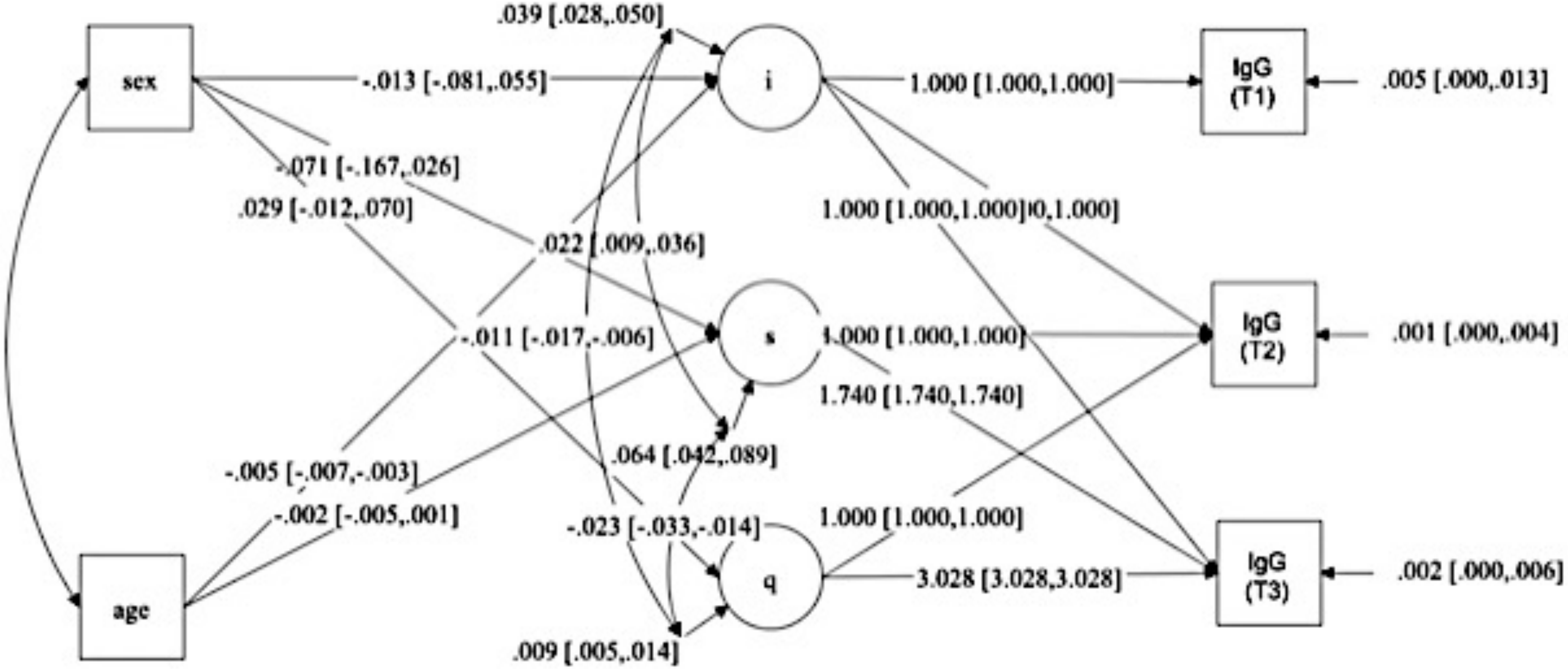

Figure S2. Latent growth curve model to determine the covariant factors on peak titer and antibody kinetics as a decreasing slope for IgG against the S protein.
i; intercept, s; slop, q; quadratic, T1; Time 1, T2; Time 2, T3; Time 3
